# A novel L1C.5 RFLP-1-4-4 recombinant porcine reproductive and respiratory syndrome virus between wild-type virus and a modified-live virus vaccine is highly pathogenic to piglets

**DOI:** 10.3389/fvets.2025.1627238

**Published:** 2025-07-02

**Authors:** Xue Gao, Jun Zhou, Runmin Kang, Haohao Lu, Shuo Feng, Yiwen Pei, Jie Liu, Zhidong Zhang, Long Zhou

**Affiliations:** ^1^Key Laboratory of Veterinary Medicine in Universities of Sichuan Province, College of Animal and Veterinary Sciences, Southwest Minzu University, Chengdu, China; ^2^Sichuan Boce Testing Technology Co., Ltd., Chengdu, China; ^3^Sichuan Provincial Key Laboratory of Animal Breeding and Genetics, Sichuan Animal Science Academy, Chengdu, Sichuan, China; ^4^Key Laboratory of Ministry of Education and Sichuan Province for Qinghai-Tibetan Plateau Animal Genetic Resource Reservation and Utilization, Chengdu, China

**Keywords:** porcine reproductive and respiratory syndrome virus, L1C RFLP-1-4-4, highly pathogenic, recombination, MLV

## Abstract

Porcine reproductive and respiratory syndrome (PRRS) is a severe viral disease in pigs caused by porcine reproductive and respiratory syndrome virus (PRRSV). It poses a severe threat to the global swine breeding industry. Recently, the emergence of PRRSV lineage 1C.5 in the United States and China with the RFLP 1–4-4 pattern has raised worldwide attention; however, there are few studies on the genome and pathogenic characteristics of the L1C.5 RFLP 1–4-4 PRRSV in China. In this study, a novel PRRSV-2 variant, designated GX2024, was isolated from a RespPRRS MLV-vaccinated piglet in China using Marc-145 cells and porcine alveolar macrophages (PAMs). The complete viral genome was further determined and analyzed. Phylogenetic analysis of its ORF5 gene showed that GX2024 belonged to PRRSV-2 L1C.5 (RFLP-1-4-4) group, whereas the complete genome sequence clustered into L8E (JXA1-like) group and it contains a discontinuous 131-aa deletion in NSP2 when compared to the NADC30 strain. Notably, recombination analyses indicated that GX2024 is a multiple recombinant virus from two wild-type PRRSVs L1C.5 (NADC30-like) and L8E (JXA1-like), and a RespPRRS MLV vaccine (L5A) strain. To tested the pathogenicity of GX2024, nine four-week-old piglets were divided into two groups (GX2024-challenge group, *n* = 5; negative control, *n* = 4). GX2024 infection caused high fever (40–42°C) and severe hemorrhagic pneumonia with pulmonary edema. The lymph nodes exhibited obvious hemorrhagic spots with lymphadenopathy. Of note, all GX2024-infected piglets died within 14 days with 100% mortality, indicating that GX2024 is a highly pathogenic PRRSV strain. Our study reports the emergence of a novel highly pathogenic L1C.5 RFLP-1-4-4 recombinant strain, which merits special attention in control and vaccine strategies in China.

## 1 Introduction

Porcine reproductive and respiratory syndrome (PRRS) is a highly contagious disease of pigs of all ages, posing serious threats to the global swine breeding industry (1). It is caused by the porcine reproductive and respiratory syndrome virus (PRRSV) belonging to the order *Nidovirales*, family *Arteriviridae*, genus *Betaarterivirus*, and is classified into two distinct species: *Betaarterivirus suid 1* (former PRRSV-1) and *Betaarterivirus suid 2* (former PRRSV-2), which exhibit a divergence of approximately 60% at the nucleotide level (2). PRRSV is an enveloped, positive-sense, single-stranded RNA virus with a 15.0–15.5 kb genome (3). Currently, 11 open reading frames (ORFs), including ORF1a, ORF1b, ORF2a, ORF2b, ORF3-7, ORF5a, and NSP2 (TF), were identified in the PRRSV genome (4). Clinically, the disease is mainly characterized by respiratory symptoms in piglets, and abortion, reproductive failure, and fetal death in pregnant sows (5, 6).

PRRSV-2 first emerged in North America in 1987 and was gradually reported in many Asian and European countries. PRRSV-2 was first identified in China in late 1995 (7), and has been widely prevalent for decades. Based on the global ORF5 gene sequences and phylogenetic classification proposed by Yim-im et al., PRRSV-2 strains were classified into 11 genetic lineages (L1–L11) with 21 sublineages in each lineage (8). Most PRRSV-2 strains in mainland China were classified as L8E (HP-PRRSV-like and CH-1a-like strains), L1C (NADC30-like and NADC34-like strains), L3 (QYYZ-like strains), and L5A (VR2332-like strains). The virulence varies among different types and lineages of PRRSV-2 strains. Currently, the L1C (NADC30-like) strain is the dominant strain in the field in China (9). The PRRSV L1C.5 with the RFLP 1-4-4 pattern outbreak in the United States in 2020, causing obvious respiratory symptoms in nursery piglets with a mortality rate of 17.5%, raised worldwide attention (10). Meanwhile, numerous RFLP 1-4-4 L1C strains were identified from 17 provinces in China from 2016 to 2021 (11), suggesting that RFLP 1-4-4 is the main pattern in circulating PRRSV strains in both the USA and China.

Genome mutations, indels, and recombination are the primary mechanisms contributing to the genetic evolution of PRRSV variants (12–15). In 2006, the Chinese highly pathogenic PRRSV (HP-PRRSV) variants (JXA1-like) were characterized by a discontinuous 30 amino acid deletion in the nsp2 coding region and caused high morbidity (50–100%) and mortality (20–100%) in infected pigs (16). In 2008, a moderately virulent PRRSV strain NADC30, featured by a discontinuous 131 amino acid deletion in the NSP2, was identified in the United States of America. In the past 2 years, the NADC30-like PRRSV was imported into China and it has become the dominant circulating strain in the field in recent years. Since the emergence of the NADC30-like strain in China, there have been an increasing number of reports of recombination events between NADC30-like and other PRRSV strains including JXA1-like (17–19), VR2332-like, QYYZ-like, and NADC34-like, adding to the complexity and the genetic diversity of PRRSV in China.

In this study, a novel recombinant PRRSV-2 strain, GX2024, was isolated from a pig farm that experienced abortion and stillbirths of pregnant sows and death of piglets in the Sichuan Province, China, in 2024. Notably, these animals have been vaccinated with an attenuated vaccine (Ingelvac® PRRS MLV), developed from the North American strain, VR-2332 (GenBank Accession No.: AY150564). The genomic features of GX2024, and its pathogenicity in piglets were further evaluated.

## 2 Manuscript and methods

### 2.1 Farm information and sample collection

In 2024, pigs (Duroc) on a farm in Sichuan province experienced abortion and stillbirths of pregnant sows, with abortion rate of ~23% (23/98). The affected 310 piglets exhibited severe clinical respiratory illness, with approximately 90% morbidity (279/310) and 50% mortality (155/310). There are no neighboring pig farms within 10 km and no similar production performance was reported around this pig farm. The sows were vaccinated with the PRRS modified-live virus (MLV) vaccine (Ingelvac® PRRS MLV) before breeding, and the piglets were vaccinated at 21 days after birth. A total of 20 lung samples of the diseased piglets were collected and sent for PRRSV detection.

### 2.2 Viral isolation and genomic sequencing

Tissue samples were homogenized and diluted with RPMI-1640 medium, the supernatant was collected after centrifugation at 10000 × *g* for 10 min at 4°C to remove residual tissue debris. The remaining lung tissues were stored in a refrigerator at −80°C. The lung samples were processed to detect PRRSV by reverse transcription polymerase chain reaction (RT-PCR). Viral RNA was extracted from the supernatants of each lung homogenate using the TRIzol reagent (Invitrogen, Carlsbad, CA, USA). The positive lung samples were used to isolate viruses from Marc-145 cells and immortalized porcine alveolar macrophages (iPAMs). The cells were maintained in RPMI-1640 medium supplemented with 10% fetal bovine serum (VivaCell, Shanghai, China) at 37°C in a humidified 5% CO_2_ atmosphere as described previously. Cell cultures were monitored every day for a cytopathic effect (CPE) (11). Indirect immunofluorescence assay (IFA) was performed to identify PRRSV using a specific monoclonal antibody targeting the PRRSV N protein (GeneTex Inc., Irvine, CA, USA). The virus was further purified by plaque assay as previously described (20). The complete genome of the PRRSV isolate was further amplified using 13 primer pairs (Supplementary Table S1). The PCR products were subjected to the commercial service (Sangon, Shanghai, China) using the Sanger sequencing, and the SeqMan program in Lasergene software (DNASTAR, Madison, WI, USA) was used to assemble the full-length genomic sequences. The genomic sequences of the PRRSV isolate were designed as GX2024 and have been submitted to the GenBank database (GenBank Accession No: PV362838).

### 2.3 Genome alignment and phylogenetic analysis

The genomic sequence of GX2024 was analyzed using MEGA-X (v10.1.8) and compared with PRRSV-2 reference sequences (partial or complete) available in GenBank. Multiple sequence alignment was carried out using the MegAlign program in Lasergene software version 7.1.0 (DNASTAR, USA). The phylogenetic analysis was conducted by using the maximum likelihood (ML) method using MEGA-X with the nucleotide substitution model GTR + G + I. Bootstrap values were calculated for 1,000 replicates of the alignment. Sequences were aligned using the ClustalW multiple alignment algorithm. The genetic lineage classification of PRRSV-2 strains follows the latest classification system described by Yim-im et al. (8).

### 2.4 Recombinant analysis

The recombination pattern of GX2024 was analyzed using the Simplot software (version 3.5.1) and the recombination detection program 4 (RDP4, version 4.96), as previously described (3). Specifically, three representative PRRSV-2 sequences of different lineages, including NADC30 (L1C), RespPRRS MLV (L5), JXA1 (L8E), and GX2024, were aligned using the MEGA-X software. The identification of possible recombination events within the genome of GX2024 were performed using seven methods, including RDP, BOOTSCAN, MAXCHI, CHIMAERA, 3SEQ, GENECONV, and SISCAN, with default settings in RDP4. Recombination events were considered significant (*p*-value < 1 × 10^−6^) when supported by at least five of the seven detection methods.

### 2.5 Viral pathogenicity in piglets

An animal challenge experiment was performed to evaluate the pathogenicity of the GX2024 isolate. Nine 4-week-old healthy Duroc piglets were purchased from a commercial pig farm with no PRRSV vaccination program. The piglets were confirmed to be negative for PRRSV, African swine fever virus (ASFV), classical swine fever virus (CSFV), porcine epidemic diarrhea virus (PEDV), pseudorabies virus (PRV) or porcine circovirus type 2 (PCV2) by PCR or RT-PCR, and determined to be free of antibodies to PRRSV by a commercial PRRSV antibody ELISA kit (IDEXX HerdChek ELISA, USA). All animals were randomly divided into two groups and maintained in individual biosafety rooms. The piglets in Group 1 (*n* = 5) were each intranasally (2 mL) and intramuscularly (1 mL) inoculated with 1 × 10^5.58^ TCID_50_/ml GX2024 isolate (the sixth passage in Marc-145) while the remaining piglets in Group 2 (*n* = 4) were mock infected with uninfected RPMI-1640 medium with the same dose. The experiments were approved by the Animal Ethics Committee of the College of Animal & Veterinary Sciences, Southwest Minzu University (registration protocol SMU-202401058).

After the PRRSV GX2024 inoculation, each piglet’s rectal temperature and clinical symptoms were monitored and scored daily (17). The scoring included the gross clinical, respiratory, and neurological symptom scores. The total score for each piglet represents the sum of the three data points above. Blood was collected from individual pigs periodically at 0, 3, 5, 7, 10, and 14 days post-inoculation (dpi). All the animals were humanely euthanized either at 14 dpi, and carcasses were subjected to harmless treatment. Piglets were weighed weekly after viral infection. In addition, tissue samples, including lungs and nodes were collected. Portions of tissue samples were immersed in 4% paraformaldehyde for histopathological and immunohistochemical (IHC) examinations as previously described (21). The ORF5 gene from the lungs was amplified and sequenced to confirm that it was the original virus.

### 2.6 Viral detection by quantitative RT-PCR (RT-qPCR)

Viremia and viral loads in tissues were quantified by reverse-transcription quantitative PCR (RT-qPCR) assay using a primer targeting the ORF7 of PRRSV-2 (Supplementary Table S2). A standard curve generated using a serially 10-fold diluted (10^9^ to 10^3^ copies/μL) plasmid containing the ORF7 was used for absolute quantification. Approximately (0.5 g) of tissue was homogenized for the tissue samples with 1 mL of RPMI-1640 medium. The supernatant was collected after centrifugation at 10000 × g for 10 min at 4°C to remove residual tissue debris (22). For each serum sample, 300 μL supernatant was used for viral RNA extraction using the TRIzol reagent (Invitrogen, Carlsbad, CA, USA). A reverse transcription kit was used to transcribe RNAs into cDNAs (TOYOBO, Shanghai, China). Viral RNA loads in serum and tissues were calculated according to the standard curve (23).

### 2.7 Detection of PRRSV antibodies

Serum samples collected at 0, 3, 5, 7, 10, and 14 dpi were used for PRRSV-specific antibodies using a commercially available ELISA kit (IDEXX HerdChek ELISA, USA). S/P > 0.4 was considered the threshold of serological positivity.

### 2.8 Statistical analysis

All data were expressed as means ± standard deviations (SD). Statistical significance between the two groups was ascertained by conducting *t*-tests with GraphPad Prism software version 10 (San Diego, CA, USA), where a *p*-value less than 0.05 was deemed to indicate statistical significance.

## 3 Result

### 3.1 Isolation and identification of novel NADC30-like PRRSV GX2024

**Figure 1:**
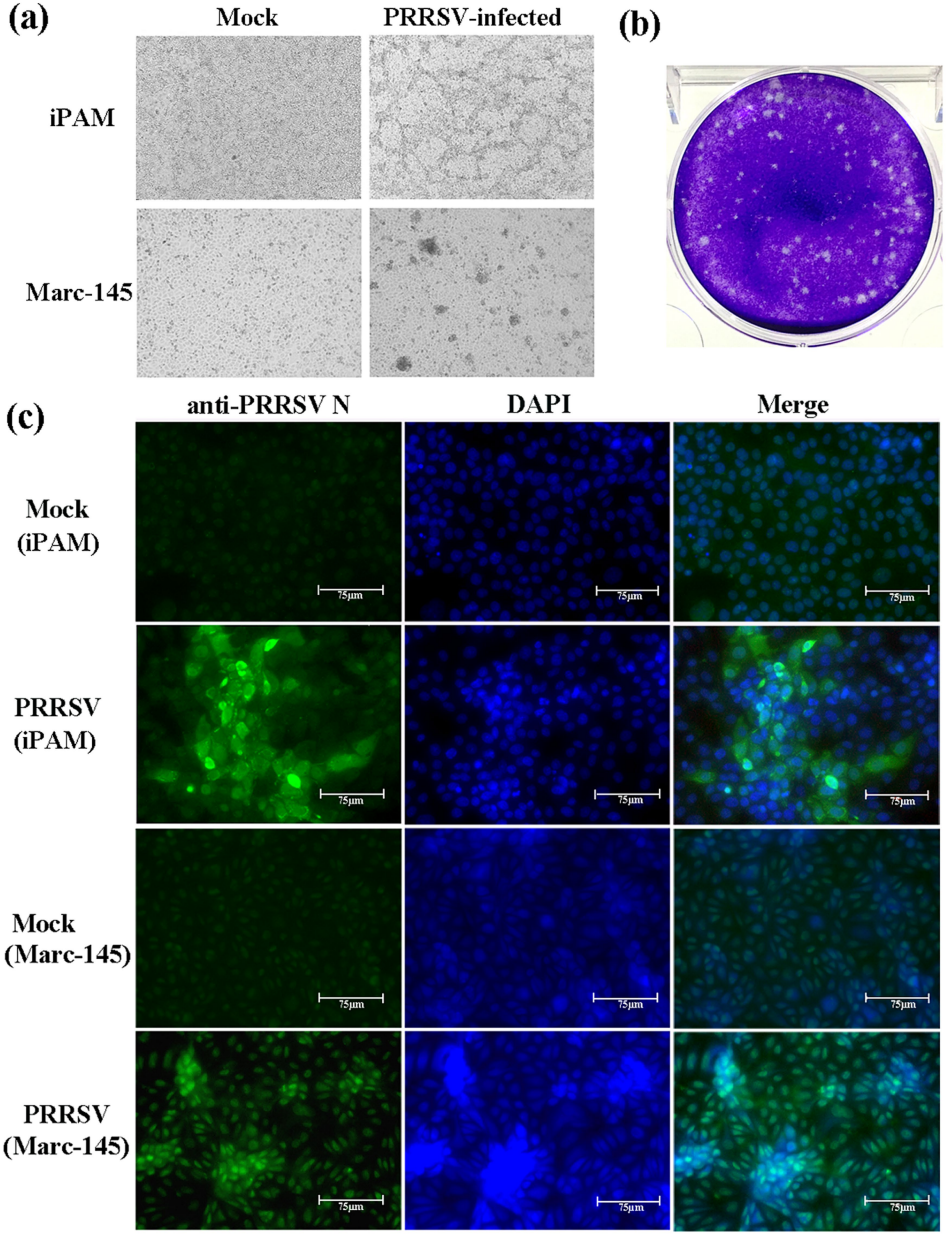
Viral isolation of PRRSV GX2024. **(a)** iPAM and Marc-145 cells were inoculated with PRRSV GX2024 as shown at 60 h postinfection; **(b)** plaque assay for GX2024 isolate with a dilution of 10^−10^ of virus stock using iPAM cells; **(c)** iPAM and Marc-145 cells were infected with six passage viral cultures for 48 h and examined by IFA with the anti-N PRRSV monoclonal antibody. Cell nuclei are stained with DAPI. Scale bar = 75 μm.

Of the 20 lung samples from the piglets with severe respiratory illness, 19 (95%) were detected PRRSV-positive by RT-PCR. The Sanger sequencing showed the sequences of Nsp2 and ORF5 among the 19 samples were identical. Therefore, GX2024 was determined to be the main causative strain of this outbreak. For viral isolation, iPAMs (immortalized PAMs)and Marc-145 were used to isolate the new PRRSV strain. The GX2024 strain was blindly passaged in iPAMs and Marc-145 cells for six passages. At 60 h after viral inoculation, the infected iPAMs and Marc-145 cells showed marked morphological changes, such as clustering and shedding. No visible CPE was observed in the uninfected cell population (Figure 1a). The viral supernatants from the sixth passage of iPAMs and Marc-145 cells were both tested using RT-PCR to confirm positivity for PRRSV. In addition, PEVD, PCV-2, PRV, and CSFV were all found to be negative by PCR or RT-PCR. We successfully isolated the GX2024 strain using a plaque forming assay and the isolated strain was confirmed by IFA experiments (Figures 1b,c). IFA was used to visualize the presence of the N protein of GX2024 in the infected iPAMs and Marc-145. Fluorescent labeling revealed anti-PRRSV N protein (green) within infected cell populations 48 h post-infection, whereas mock-infected cells showed no detectable signal under identical imaging conditions (Figure 1c). The results indicated that the GX2024 isolate could replicate in iPAM and Marc-145 cells. The TCID_50_ value of the GX2024 isolate was 1 × 10^5.58^/mL using Marc-145 cells.

### 3.2 Complete genomic characterization of the GX2024 isolate

The complete genomic sequence of GX2024 was 15,018 nucleotides (nt), not including the poly(A) tail structure. The sequences were deposited in the GenBank database under the accession number PV362838. The comparative analysis revealed that GX2024 exhibited sequence similarity levels of 82.4, 87.0, 85.5, and 81.4% when aligned with reference strains VR-2332 (L5A), JXA1 (L8E), NADC30 (L1C), and NADC34 (L1C), respectively (Table 1). Furthermore, the genome of the GX2024 isolate was compared with 10 PRRSV representative strains (VR-2332, RespPRRS MLV, JXA1, JXWn06, NADC30, CHsx1401, FJ1402, NADC34, RFLP 1–4-4, and L1C.5). The ORF1a (Nsp1, Nsp3-8) and ORF1b (Nsp9, NSP11) of GX2024 shared 89.9–100% nt similarity with JXA1-like (L8) strains, which higher than the similarity shared with other strains. Nsp2, NSP10, Nsp12, ORF2a, ORF2b and ORF4-7 of GX2024 shared 83.7–95.9% nt similarity with NADC30-like (L1) strains. The ORF3 shared the highest nt identity (90.1%) with RespPRRS MLV, higher than the similarity shared with its parent strain VR-2332 (89.8%). These findings suggest that the GX2024 isolate may have undergone chimeric recombination events.

**Table 1:** Nucleotide identity of GX2024 compared with eleven PRRSV reference strains.

**Nucleotide Identity % (GX2024)**										
**Region**	**VR2332**	**MLV**	**JXA1**	**JXWn06**	**NADC30**	**CHsx1401**	**FJ1402**	**NADC34**	**RFLP 1–4-4 L1C**	**L1C.5**
	**VR-2332-like** **(Lineage 5)**		**JXA1-like** **(Lineage 8)**		**NADC30-like** **(Lineage 1)**			**NADC34-like (Lineage 1)**	**Lineage 1** **(L1C)**	
Complete genome	82.4	82.4	87.0	**87.1**	85.5	84.3	85.2	81.4	83.5	83.3
ORF1a	81.4	81.4	89.9	**90.2**	84.8	83.5	86.0	79.7	79.6	79.3
ORF1b	88.4	88.3	91.6	**91.7**	90.8	89.6	89.0	87.4	86.9	87
nsp1	90.0	89.9	89.9	**99.4**	85.9	83.6	84.1	84.3	82.8	82.8
nsp2	68.1	68.0	75.7	75.8	**84.8**	83.7	**84.8**	73.4	70.2	70.0
nsp3	90.4	90.5	99	**99.1**	83	82.7	93.1	83	83.6	83.4
nsp4	90.2	90.2	99.4	**99.5**	85.4	84.9	84.7	85.9	87.1	85.6
nsp5	89.4	89.4	98.8	**99.6**	90.6	87.4	88.6	84.1	87.0	87.8
nsp6	93.8	93.8	**97.9**	**97.9**	91.7	81.2	93.8	85.4	95.8	91.7
nsp7	88.9	89.3	99.0	**99.2**	82.2	81.7	81.1	81.5	81.2	81.0
nsp8	96.3	96.3	99.3	**100**	89.6	89.6	88.9	91.1	87.4	83.7
nsp9	91.4	91.4	97.8	**98.0**	87.8	87.2	86.5	87.4	87.0	87.2
nsp10	85.0	84.8	83.9	84.0	**94.1**	92.3	89.8	89.2	88.3	88.5
nsp11	87.2	87.1	**92.6**	92.6	90.3	87.9	90.3	85.9	85.3	85.6
nsp12	87.6	87.6	86.3	86.3	**94.6**	93.9	86.9	84.3	84.5	84.3
ORF2a	88.3	88.1	85.5	85.5	**91.8**	90.0	85.5	85.2	83.7	82.9
ORF2b	91.4	91.0	90.1	90.1	**95.9**	94.6	89.2	89.2	87.8	86.5
ORF3	89.8	**90.1**	86.3	85.9	87.7	88.0	88.1	86.8	86	86.1
ORF4	86.8	86.8	85.7	86.2	**95.3**	92.7	93.9	93.9	91.8	91.1
ORF5	85.1	85.1	85.1	85.2	**93.5**	92.2	92.7	87.9	92.5	91.5
ORF6	89.1	89.0	89.5	89.3	**91.8**	91.0	91.0	89.1	91.4	91.4
ORF7	89.5	89.5	87.4	85.8	**92.5**	91.1	91.9	91.7	90.6	90.6

Bold face numbers depict the highest percentage identity.

### 3.3 Phylogenetic analysis

**Figure 2:**
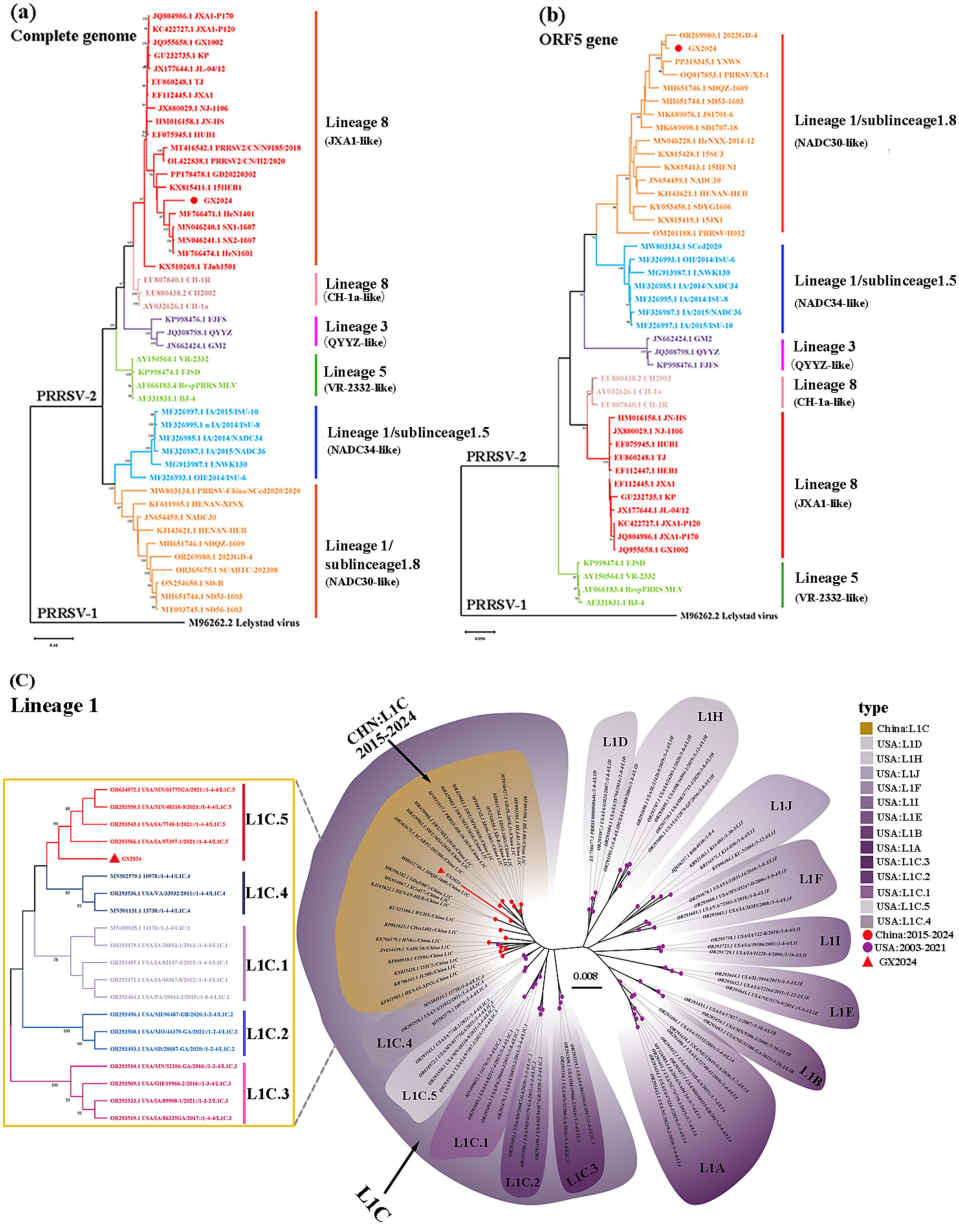
Phylogenetic analysis of GX2024. **(a)** Phylogenetic tree based on the whole genome of GX2024 virus strain; **(b)** phylogenetic tree based on ORF5 sequence; **(c)** phylogenetic tree based on ORF5 nucleotides of PRRSV-2 lineage 1 strains. The GX2024 isolate in this study are labeled with “red triangle.” The phylogenetic tree was constructed by the maximum likelihood method in the MEGA-X software and the bootstrap values of 1,000 replicates were calculated.

In order to determine the genetic relationship between GX2024 and other PRRSV strains, ML phylogenetic trees based on complete genome and ORF5 sequences were generated using MEGA-X software (Figure 2). According to ORF5 genotyping, all PRRSV-2 strains in China belonged to one of the four lineages: L8E (JXA1-/CH-1a-like), L1C (NADC30-/NADC34-like), L5A (VR-2332-like), and L3 (QYYZ-like). The GX2024 isolate was classified in L8E based on its whole genomic sequences (Figure 2a), whereas it belonged to L1 based on ORF5 genotyping (Figure 2b), suggesting that GX2024 may be a recombinant strain. Furthermore, the L1C phylogenetic tree showed that the L1C strains in the USA displayed more abundant genetic diversity than those in China and the GX2024 clustered with L1C.5 strains with RFLP 1–4-4 pattern from the China during 2015–2024 (Figure 2c). These findings indicated that the L1C PRRSVs in China and the USA undergone distinct genetic evolution processes.

### 3.4 Amino acid analysis of Nsp2 and GP5

**Figure 3:**
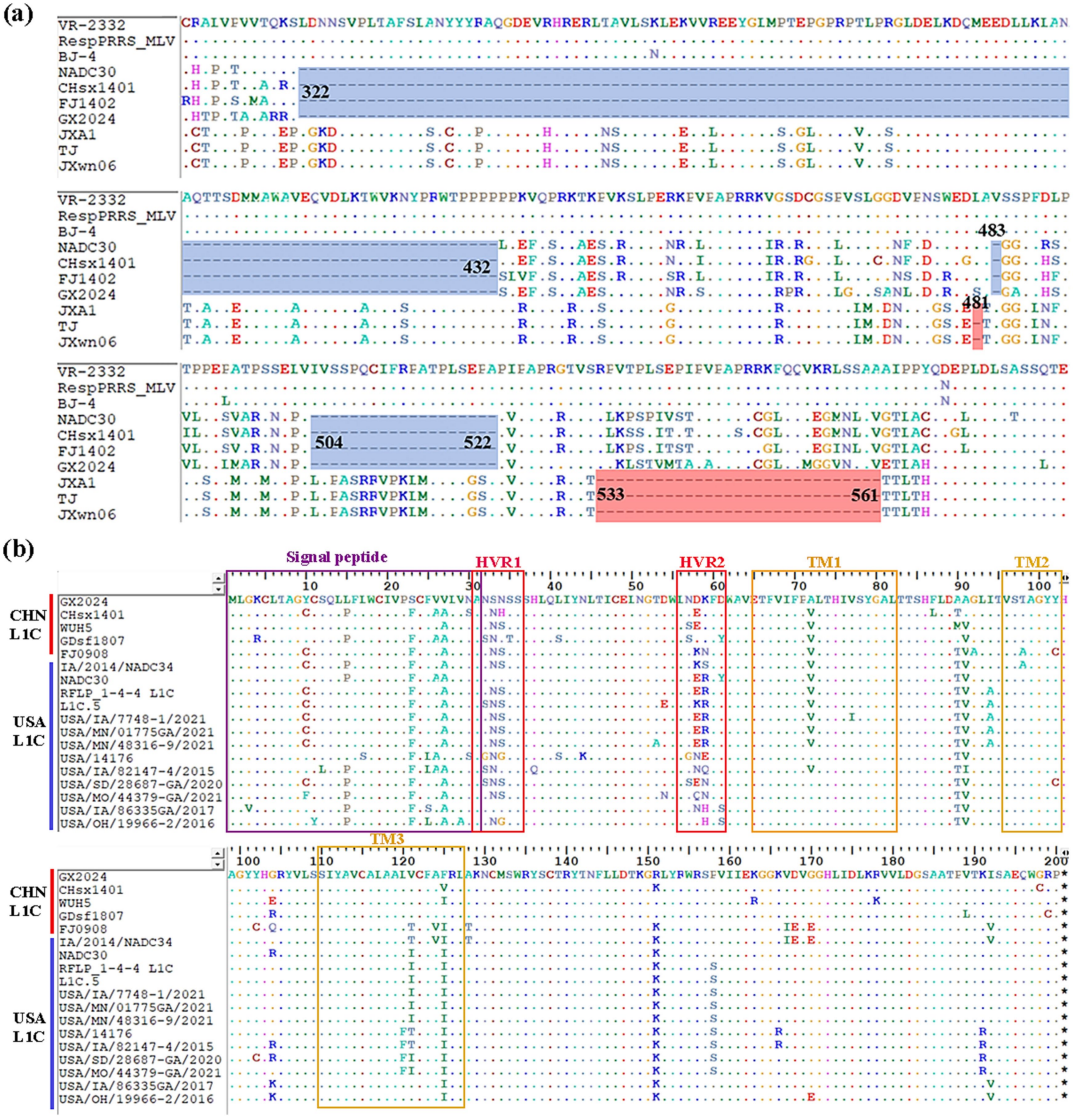
Multiple amino acid sequences alignment of NSP2 and GP5. **(a)** Three discontinuous amino acid deletions at positions 322–432, 483, 504–522 (blue regions) in NSP2 of GX2024 and NADC30-like strains. The light red regions indicate the amino acid deletions of Chinese HP-PRRSV (JXA1-like) strains. **(b)** Multiple comparisons of GP5 amino acid sequences from GX2024 and 17 L1C PRRSV reference strains from China and the USA. Purple box indicates signal peptide region, red boxes indicate highly variable regions (HVR1 and 2), and yellow boxes indicate transmembrane structural domains (TM1, 2, and 3).

Comparison of the NSP2 HV region in GX2024 and other reference strains revealed that the GX2024 isolate shared identical noncontiguous amino acid deletions (111 + 1 + 19-aa), 111-aa deletion at positions 322–432, 1-aa deletion at position 483, and 19-aa deletion at positions 504–522 with the L1 representative strain NADC30, which was reported in the United States in 2008 (Figure 3a). In addition, comparisons of the amino acid analyses of GX2024 GP5 with those of the other L1C strains from China and the United States showed distinct amino acid mutation sites, such as A^94^, F^120^, S^158^, and R^191^, were identified in L1C strains from the USA. Notably, GX2024 isolate exhibited several unique amino acid substitutions, including P^15^ → L^15^, F^23^ → S^23^, A^26^ → V^26^, and A^27^ → V^27^ (Figure 3b).

### 3.5 Recombination analysis

**Figure 4:**
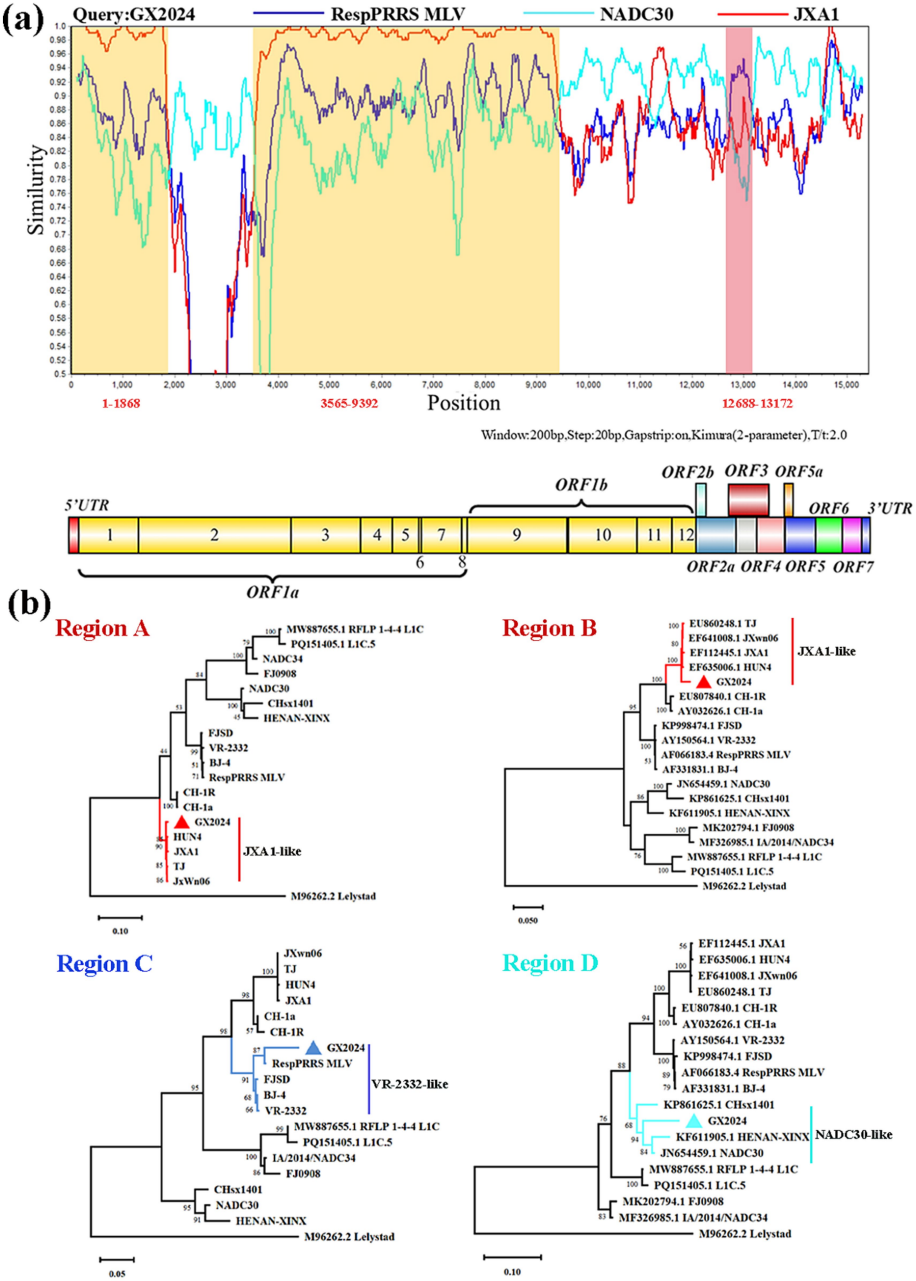
Genome recombination analysis of GX2024 isolate. **(a)** The *y*-axis indicates the percentage similarity between the query sequence (GX2024) and three representative sequences. Genome scale similarity comparisons of GX2024 (query) with NADC30 (Cyan), JXA1 (red), and RespPRRS MLV (blue). The supposed recombination regions are shown with two yellow shadows and a Light red shadow. The recombination breakpoints are marked at the bottom with nucleotide sites and viral genome structure referenced to VR-2332. **(b)** Phylogenetic trees based on each recombinant fragment (Regions A-D) of GX2024 was constructed to confirm the accuracy of recombination events. Region A and B indicate the nucleotide sequences of yellow shadow, region C indicates the nucleotide sequences of Light red shadow, and the region D indicates the nucleotide sequences of white regions. The GX2024 isolate in this study is labeled with “triangle”.

We examined the sequence alignment between GX2024 and reference PRRSV strains using RDP4 and SimPlot to identify potential recombination events. RDP4 analysis revealed that GX2024 is a natural recombinant virus derived from three parental strains with *p*-values of ≤ 4.264 × 10^−9^, from the results of at least six detection methods (Table 2). The NADC30 was the major parental strain, and the JXA1 and RespPRRS MLV were the minor parental strains. Similarity plotting identified five recombination breakpoints in the GX2024 genome. They were located in ORF1a (nt 1868, 3,565), ORF1b (nt 9,392), ORF3 (nt 12,688), and ORF4 (nt 13,172) (Figure 4a). The five breakpoints separated the genome into six regions, with region A (nt 1–1868) and region B (nt 3,565–9,392) being closely related to the JXA1-like strain (L8), and region C (nt 12,688-13,172) being closely related to the RespPRRS MLV strain (L5). Region D (nt 1869–3,564, 9,393–12,687 and 13,173–3’-UTR) was classified into the NADC30-like cluster (L1) (Figure 4b). Therefore, the naturally recombinant virus GX2024 identified in this study was derived from the recombination of lineage 1 (NADC30-like), lineage 8 (JXA1-like), and the attenuated vaccine strain RespPRRS MLV (lineage 5).

**Table 2:** Information on recombination events detected in GX2024.

Recombinant strain	Breakpoints		Parental Sequence		Detection methods (*p*-value)						
	Beginning	Ending	Major	Minor	RDP	GENECONV	BootScan	MaxChi	Chimaera	SiScan	3Seq
**GX2024**	1	1868	JXA1	NADC30	7.009 × 10^−106^	8.600 × 10^−118^	7.416 × 10^−117^	2.907 × 10^−35^	2.307 × 10^−35^	4.104 × 10^−43^	8.881 × 10^−16^
	3,565	9,392	JXA1	NADC30	2.458 × 10^−158^	8.520 × 10^−162^	7.273 × 10^−165^	4.379 × 10^−60^	1.030 × 10^−49^	2.383 × 10^−83^	NS
	12,688	13,172	RespPRRS MLV	NADC30	3.536 × 10^−26^	1.057 × 10^−14^	5.790 × 10^−24^	7.775 × 10^−12^	NS	3.110 × 10^−11^	4.264 × 10^−9^

NS: not significant.

### 3.6 Pathogenicity of the NADC30-like PRRSV strain GX2024 in piglets

#### 3.6.1 Clinical symptoms

**Figure 5:**
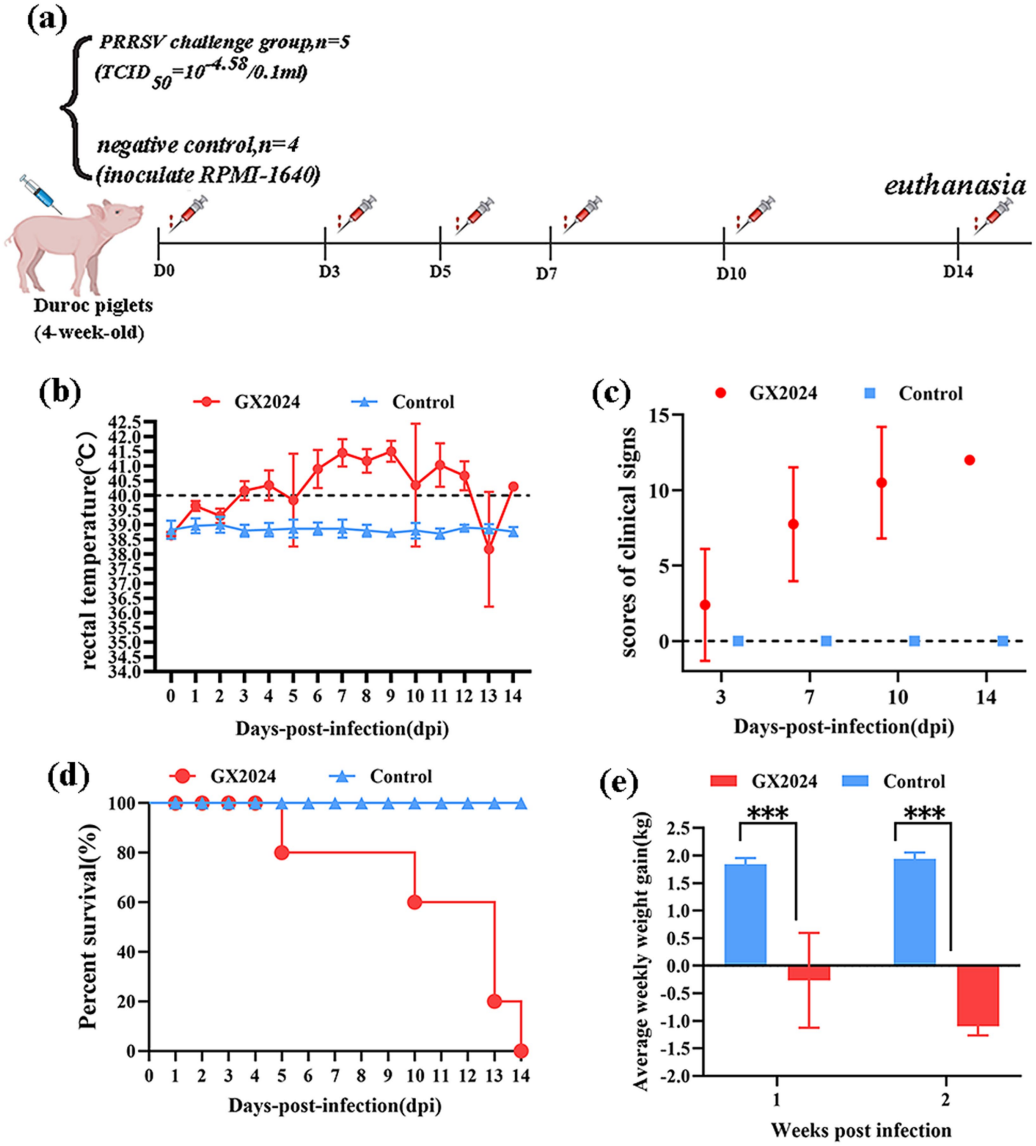
Pathogenicity analysis of the PRRSV isolate GX2024 in piglets. **(a)** Animal experimental design of this study. **(b)** Rectal temperatures of pigs inoculated with GX2024 and uninfected RPMI-1640 medium. The clinical fever cut-off value was set at 40.0°C. **(c)** The scores of clinical signs at 3, 7, 10, 14 dpi of the challenge study. The scoring included the gross clinical, respiratory, and neurological symptom scores. **(d)** The survival and mortality curves of the inoculated pigs. **(e)** Average weekly weight gain of the inoculated pigs during the challenge experiment. The measured values in this study were expressed as the mean ± standard deviations (SD). Asterisk (*) indicates significant differences between the GX2024 and RPMI-1640 medium-inoculated groups (*p < 0.05; **p < 0.01; ***p < 0.001).

To evaluate the pathogenicity of the GX2024 isolate, five 4-week-old piglets in the PRRSV challenge group were each intranasally (2 mL/piglet) and intramuscularly (1 mL/piglet) inoculated with 1 × 10^5.58^ TCID_50/_ml GX2024 isolate, and four piglets in the negative control group were inoculated with uninfected RPMI-1640 (Figure 5a). During the challenge period, the piglets in the GX2024-challenge group began to show a febrile response (above 40.0°C) at 3 dpi, and exhibited slight clinical signs within 1–3 dpi, such as anorexia, coughing, sneezing, vomiting, and delayed mobility. More severe clinical manifestations, including high fever (the highest up to 42°C), respiratory distress, shivering, ataxia, and diarrhea, were manifested within 4–14 dpi (Figure 5b). The average clinical scores of the inoculated pigs were significantly higher than those of the RPMI-1640-inoculated group (*p* < 0.05) starting from 3 dpi until the end of the experiment (Figure 5c). After the inoculation of GX2024, four piglets died at 5–13 dpi with red and bloody nasal discharge; the remaining one in the challenge group was moribund and euthanized (humane endpoints) at 14 dpi (Figure 5d). The body weights of the GX2024-inoculated pigs decreased significantly and showed negative growth 1–2 weeks post-infection (Figure 5e). The average rectal temperature of the RPMI-1640-inoculated pigs remained below 40°C and exhibited no obvious clinical signs throughout the experiment (Figures 5b–e).

#### 3.6.2 Viral loads in serum and tissues, and PRRSV-specific antibodies

**Figure 6:**
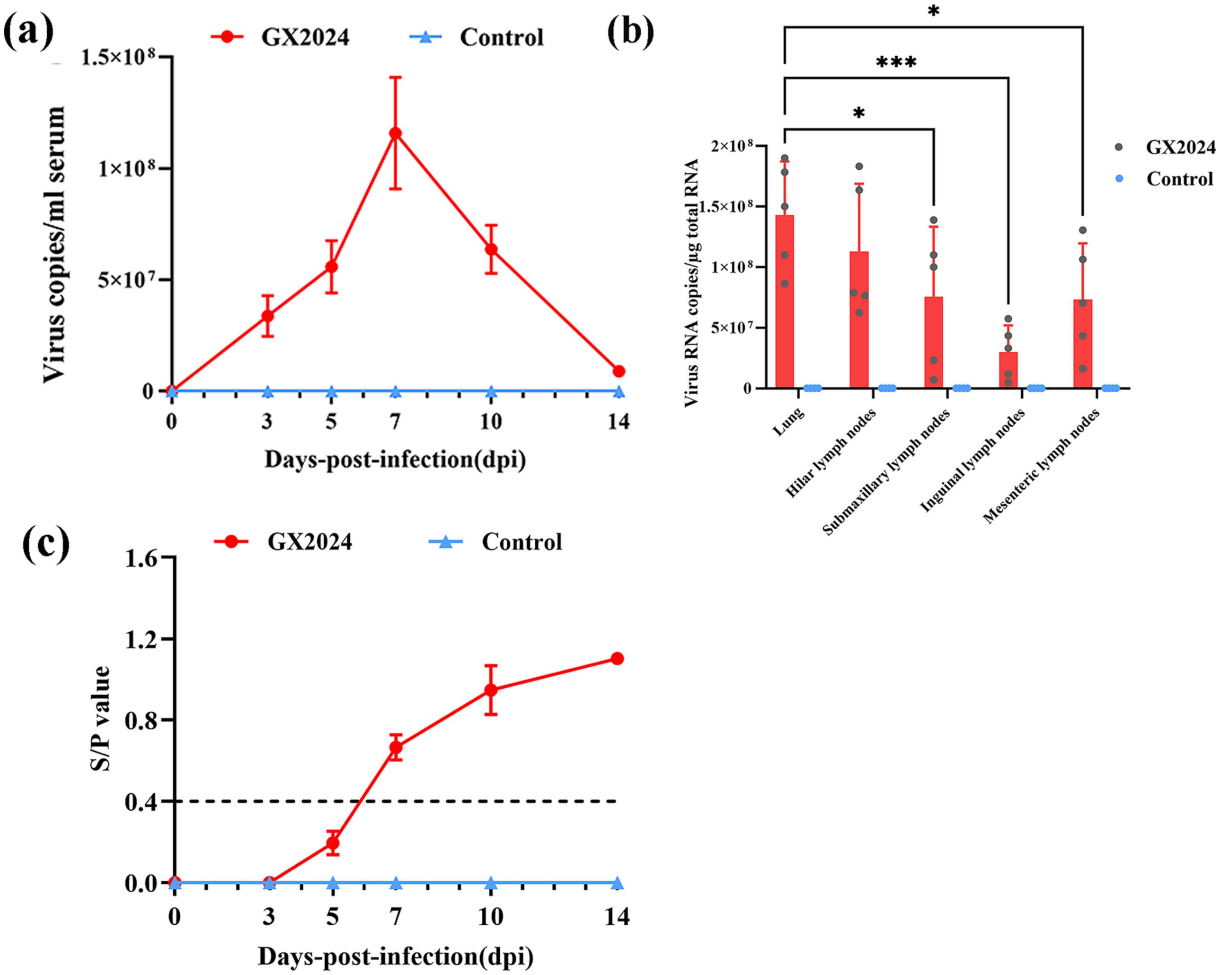
Detection of viral loads in serum and tissues, and PRRSV-Specific Antibodies. **(a)** The PRRSV RNA copy numbers in serum of inoculated pigs at different days post challenge were detected by qRT-PCR. **(b)** The PRRSV RNA copy numbers in tissues of inoculated pigs at post challenge were detected by qRT-PCR. The lungs exhibited significantly higher viral loads than submaxillary lymph nodes (*p* < 0.05), inguinal lymph nodes (*p* < 0.001) and mesenteric lymph nodes (*p* < 0.05) (∗*p* < 0.05; ∗∗*p* < 0.01; ∗∗∗*p* < 0.001). **(c)** PRRSV-specific antibodies in serum of challenged pigs at different days post challenge. S/P > 0.4 was considered as the threshold of serological positivity. The measured values in this study were expressed as the mean ± SD.

The viral loads in serum and tissues including the lung, hilar lymph node, submaxillary lymph node, inguinal lymph node, and mesenteric lymph node were determined. As shown in Figure 6, the serum virus RNA copy numbers in the GX2024-infected piglets increased rapidly at 3 dpi, and reached a peak at 7 dpi, followed by a rapid decrease (Figure 6a). The viral load measurement in tissues showed that the lungs exhibited significantly higher viral loads than submaxillary lymph nodes (*p* < 0.05), inguinal lymph nodes (*p* < 0.001) and mesenteric lymph nodes (*p* < 0.05).(Figure 6b). In addition, serum samples were collected for PRRSV N protein antibody measurements. In the GX2024-inoculated group, all piglets were seropositive (S/P > 0.4) at 7 dpi. Subsequently, the levels of PRRSV antibodies gradually increased. In particular, the mean S/P value reached about 1.1 at 14 dpi, whereas the control group was consistently negative (*S*/P < 0.4) throughout the experiment (Figure 6c).

#### 3.6.3 Pathological examination and immunohistochemistry

**Figure 7:**
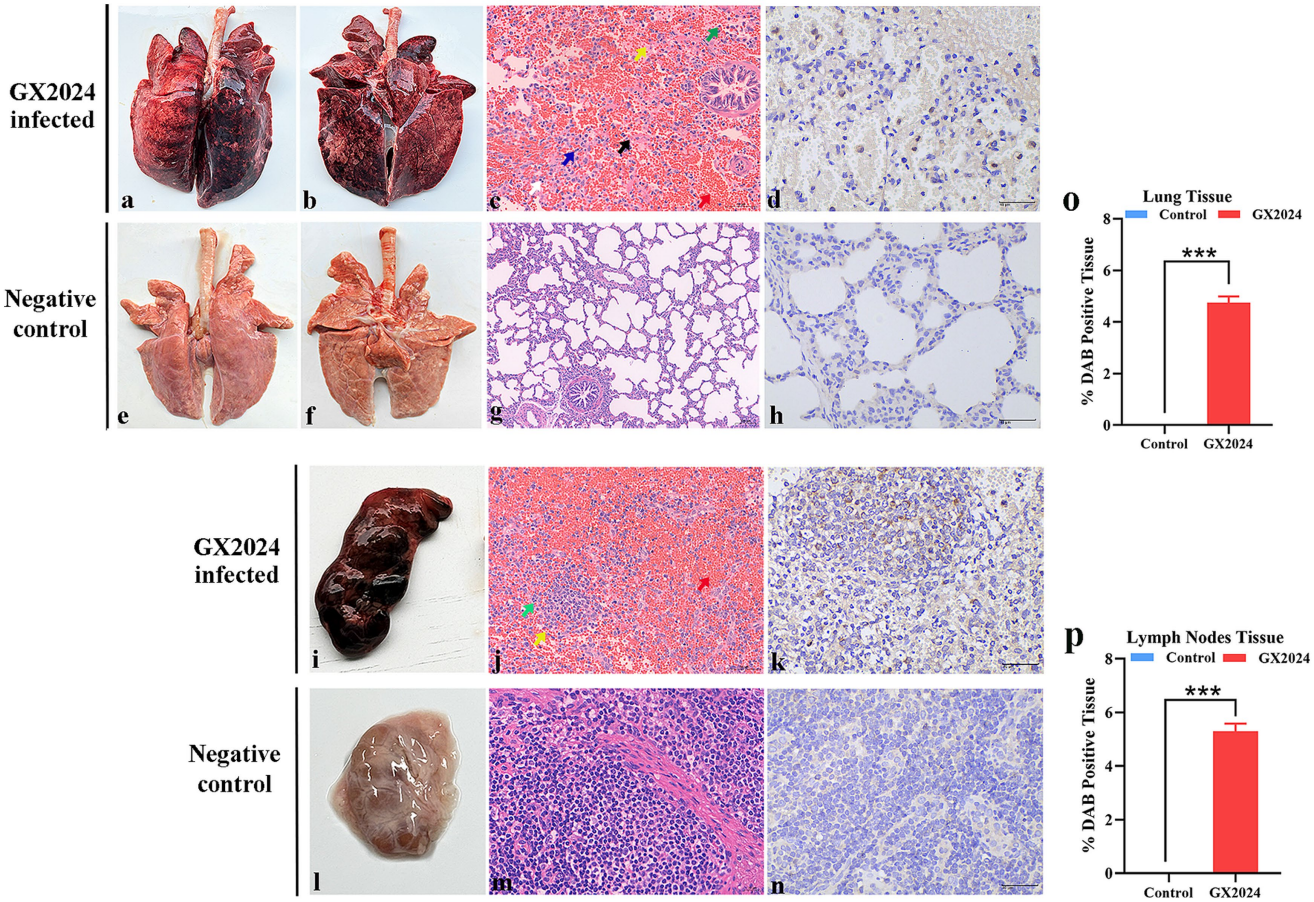
Lungs and lymph nodes lesions observation of the inoculated piglets. **(a,b)** Severe hemorrhagic pneumonia in dorsal and ventral sides of lung with pulmonary consolidation and edema were observed in GX2024-inoculated pigs. **(e,f)** No gross lung changes were observed in the negative control pigs. **(c)** Interstitial pneumonia characterized by marked thickening of the alveolar septa, the hyperplasia and necrosis of the alveolar epithelium (black arrow), and severe inflammation characterized by infiltrating neutrophils (green arrow) and lymphocytes (white arrow) were observed in GX2024-inoculated pigs. Fibrous tissue hyperplasia and a large number of red blood cells were observed in the lung stroma (red arrow). **(g)** No microscopic lesions were observed in the control piglets. **(d)** PRRSV-specific antigen positive signals mainly distributed in a cytoplasm of macrophages in the within the alveolar wall cells. **(h)** No PRRSV-positive signals was observed in the control pigs. **(i)** The lymph nodes of pigs inoculated with the GX2024 strain exhibited swelling and severe hemorrhage. **(l)** No gross lymph nodes changes were observed in the RPMI-1640-inoculated pigs. **(j)** The GX2024-inoculated group displayed an unclear boundary between the cortex and medulla. Numerous lymphocytes were decreased (yellow arrow), a small part of lymphocytes exhibited necrosis (green arrow), and massive red blood cells were observed in cortical and subcortical areas (red arrow). **(m)** No microscopic lesions were observed in the control piglets. **(k)** PRRSV-specific antigen positive signals were detected in the lymph nodes of GX2024-infected pigs. **(n)** No PRRSV-positive signal was observed in the control piglets. Original magnification, 400×. **(o)** The PRRSV-positive lung tissue ratio of lung in the GX2024-challenge were significantly higher than the control group (*p* < 0.001). **(p)** The PRRSV-positive lung tissue ratio of lymph nodes in the GX2024-challenge were significantly higher than the control group (*p* < 0.001). The *** means *p* < 0.001.

At 14 dpi, all the pigs were euthanized and necropsied. The major pathological lesions in the GX2024-chanllenge group were characterized by pulmonary consolidation, edema, and severe hemorrhage in the dorsal and ventral sides of the lungs (Figures 7a,b). The lymph nodes exhibited swelling and severe hemorrhage (Figure 7i). No noticeable macroscopic lesions were found in the lungs and lymph nodes of the negative control group (Figures 7e,f,l). Using H&E staining and microscopic histopathological examinations showed that all the GX2024-challenged pigs developed interstitial pneumonia characterized by marked thickening of the alveolar septa, the hyperplasia and necrosis of the alveolar epithelium, and severe inflammation characterized by infiltrating neutrophils and lymphocytes. Fibrous tissue hyperplasia and significant red blood cells were observed in the lung stroma (Figure 7c). The lymph nodes in the GX2024-inoculated group displayed an unclear boundary between the cortex and medulla. Numerous lymphocytes were decreased, a small part of the lymphocytes exhibited necrosis, and massive red blood cells were observed in the cortical and subcortical areas (Figure 7j). Normal histology was determined in the lymph nodes of the negative control group (Figures 7g,m). PRRSV-specific IHC staining was also performed on serial sections of all groups’ lungs and lymph nodes. PRRSV-specific antigen-positive signals were detected in the lungs (Figure 7d) and lymph nodes (Figure 7k) of GX2024-infected pigs, and no positive signals were detected in the negative control group (Figures 7h,n). Statistical analysis showed that the PRRSV-positive tissue ratio of lung and lymph nodes in GX2024-challenge was significantly higher than the control group (*p* < 0.001) (Figures 7o,p).

## 4 Discussion

Since the outbreak of highly pathogenic PRRS in China in 2006, the HP-PRRSV-like strains have had a long-lasting impact on the Chinese swine industry (24). Despite several HP-PRRSV-derived attenuated live vaccines, such as JXA1-P80, TJM-F92, HuN4-F112, R98, and GDr180 (25), which have been widely used in the field, the disease still cannot be completely controlled. With the importation of lineage 1 of PRRSV-2 strain represented by NADC30 from the United States, the prevalence status has gradually changed in China. At present, the NADC30-like strains have widely spread in China and become locally dominant viruses in many provinces (26–29). By phylogenetic analysis, L1C strains with RFLP 1-4-4 displayed the most common pattern in circulating PRRSV strains in China, which is the same with L1 strains in the United States (10); however, the L1C.5 strains in China and the USA were clustered into two instinct branches, suggesting that these strains have undergone different process of genetic evolution in the two countries.

Due to the coexistence of multiple lineages of PRRSVs in China, these strains face a greater risk of genomic recombination (30). To date, numerous recombinant strains between L1 and other lineages, including L8, L3, and L5 have been reported in China (31). In this study, GX2024 is a recombinant strain among NADC30-like PRRSV (L1C.5), HP-PRRSV JXA1-like (L8E) and a RespPRRS MLV vaccine (L5A). A partial ORF3 was derived from the RespPRRS MLV vaccine strain in GX2024. Because the wild-type and RespPRRS MLV strain and its parent strain VR-2332 share high identity, we further confirmed that GX2024 is a recombinant strain of RespPRRS MLV vaccine and not VR-2332 wild-type because of the higher nucleotide similarity to RespPRRS MLV in GX2024. Our results indicated that the L1C.5 PRRSV strains in China were confronted with complicated and diversified circumstances.

Recombination is an important mechanism of PRRSV evolution (32). Our previous study reported a natural recombinant PRRSV strain SCN17, which shared the same recombination pattern (NADC30-like + JXA1-like + RespPRRS MLV) with GX2024. Animal challenge experiments with piglets showed that SCN17 is a moderately virulent strain with mild clinical presentations, and all SCN17-infected piglets survived throughout the study (3). In this study, GX2024 infection caused high fever (the highest up to 42°C) and severe hemorrhagic pneumonia with pulmonary edema. The lymph nodes exhibited obvious bleeding points with lymphadenopathy. Of note, all GX2024-infected piglets died within 14 days with 100% mortality (5/5), indicating that GX2024 is a highly pathogenic PRRSV strain. We speculate that the distinct pathogenicity in pigs might be related to the different recombination regions of the two strains. For SCN17, NADC30-like virus (moderately virulent strain) and RespPRRS MLV are the major parent strains, whereas HP-PRRSV JXA1 and L1C.5 is the major parent strains of GX2024. Recently, a pathogenicity test for piglets revealed that the PRRSV 1–4-4 L1C variant (L1C.5) is highly virulent than L1C.1, L1A, L1H and 1-7-4 L1A isolates (10). Therefore, the uptake of nucleotide sequences from HP-PRRSV JXA1-like strain and the highly virulent L1C.5 strain by the genome of GX2024 might lead to elevated virulence of the new isolate. In China, a recombinant variant of PRRSV with RFLP 1-4-4 and L1C features, HuN2021, which recombined with HP-PRRSV JXA1-like strain, was demonstrated to be a highly pathogenic strain in experiments in piglets (11). Thus, recombination occurring among L1C.5 PRRSV strains could lead to the emergence of novel and more virulent viruses.

Vaccination is thought to be an effective method for controlling and preventing HP-PRRSV (JXA1-like, L8E) in China (33, 34). However, prevention and control of the current L1C PRRSV pandemic still face enormous challenges, such as limited heterologous cross-protection between different lineage strains (5, 35–37), risks of virulence reversion of vaccine strains (38), and recombination among PRRSV field strains as well as between field and MLV vaccine strains (39). In this study, GX2024 exhibits immunological escape from the currently available RespPRRS MLV vaccine, and recombination with the vaccine strain occurred. Thus, the L1C PRRSVs in China merit special attention when devising control and vaccine strategies, and increasing biosafety measures and viral eradication in pig farms, together with the use of antiviral drugs may be an effective way to control PRRS.

## 5 Conclusion

In conclusion, a novel L1C.5 RFLP-1-4-4 variant of PRRSV, GX2024, was isolated and its whole genome was sequenced and characterized. GX2024 is a recombinant strain among NADC30-like PRRSV (L1C.5), HP-PRRSV JXA1-like (L8E), and a RespPRRS MLV vaccine (L5A), which has never been described. Animal challenge experiments with pigs showed that GX2024 is a highly pathogenic PRRSV strain with high fever, high morbidity and mortality. This study highlights the importance of the surveillance of PRRSV with 1-4-4 and L1C characteristics in pigs and the necessity to update the vaccine strategies against newly emerging PRRSV strains in China.

## Data Availability

The datasets presented in this study can be found in online repositories. The names of the repository/repositories and accession number(s) can be found in the article/Supplementary material.
