# Supplementary material for: A novel L1C.5 RFLP-1-4-4 recombinant porcine reproductive and respiratory syndrome virus between wild-type virus and a modified-live virus vaccine is highly pathogenic to piglets
Source: Front Vet Sci. 2025 Jul 2;12:1627238. doi: 10.3389/fvets.2025.1627238 (PMC12263378; doi:10.3389/fvets.2025.1627238)
Supplement: Supplementary file 1 [file Table_1.DOCX]

Supplementary Material

## Supplementary Tables

**Table S1:** Specific primers used to amplify the genome of PRRSV GX2024 of in this study.

| Virus | Primer name ^a^ | Primers sequence (5´-3´) | Primer position | Length (bp) |
| --- | --- | --- | --- | --- |
| 1 | 1F | ATGACGTATAGGTGTTGGCTCT | 1-22 | 1573 |
|  | 1R | CTCTTTCRGGRAGGGTGGTYTC | 1552-1573 |  |
| 2 | 2F | AAYTGTGGTTGGCACTG | 1495-1511 | 708 |
|  | 2R | CCAATCAAAGGAGGTKTC | 2185-2202 |  |
| 3 | 3F | CCGCTACTACGTGGACTGTTT | 1937-1957 | 1773 |
|  | 3R | TCTGAAGACGGCAAATCAGTG | 3690-3710 |  |
| 4 | 4F | GAGCYYCTSGATTTGTCTG | 3043-3061 | 1058 |
|  | 4R | GTTCTYACACAAGATCCCCA | 4381-4400 |  |
| 5 | 5F | TGGCTGGAGCYTATGTGCTTTC | 4337-4358 | 1249 |
|  | 5R | AACARCATGGTRCGACCAGTCA | 5564-5585 |  |
| 6 | 6F | CCTGGTTGCTTTGTGTGTTTC | 5195-5215 | 1425 |
|  | 6R | GAAGAGAACACCCCGTCGCCA | 6600-6620 |  |
| 7 | 7F | TGGTYRTGACCTCRCCAGTCCCAG | 6587-6610 | 1607 |
|  | 7R | TAAGGTATGTCTCCAAACCTTGT | 8172-8194 |  |
| 8 | 8F | TTTGAGTTGTATGTGCCGACC | 8220-8240 | 1639 |
|  | 8R | CCCTGATGCCACGCCTAACGG | 9838-9858 |  |
| 9 | 9F | TGAATGGTTTGAAGAGCTCGT | 9413-9433 | 1531 |
|  | 9R | GTTCTGCCGGGAGTTTAGCAA | 10924-10944 |  |
| 10 | 10F | GTGTATGACCCACACAGGCAA | 10638-10658 | 661 |
|  | 10R | TTTGGCACCATAAGGTGTCAA | 11278-11298 |  |
| 11 | 11F | GCGAGAAGTTGCCGAGTC | 10908-10925 | 2184 |
|  | 11R | GATAGTGATGTAAACGGGTGT | 13070-13090 |  |
| 12 | 12F | GCTACACGGCCCAGTTCCATC | 12958-12978 | 1805 |
|  | 12R | TGCCACCCAACACGAGGCTTT | 14742-14762 |  |
| 13 | 13F | 5' GGCAAATGATAACCACG 3' | 14360-14376 | 659 |
|  | 13R | 5' TGACTTAGAGGCACAATAC 3' | 15000-15018 |  |

^a^ F and R represent forward and reverse primers, respectively.

**Table S2**: Information of RT-qPCR primers targeting the ORF7 of PRRSV GX2024.

| Virus | Primer name ^a^ | Primers sequence (5´-3´) | Primer position | Length (bp) |
| --- | --- | --- | --- | --- |
| GX2024 | F | GACAGTCCAGAGGCAAGG | 14596-14613 | 221 |
| GX2024 | R | TAGGCAAACTAAACTCCACA | 14798-14817 |  |
